# The effect of anxiety on working memory and language abilities in elementary schoolchildren with and without Additional Health and Developmental Needs

**DOI:** 10.3389/fpsyg.2022.1061212

**Published:** 2022-12-15

**Authors:** Hayley E. Pickering, Carl Parsons, Sheila G. Crewther

**Affiliations:** ^1^Department of Psychology, Counselling and Therapy, La Trobe University, Melbourne, VIC, Australia; ^2^SHINE Programs, Port Melbourne, VIC, Australia; ^3^Centre for Human Psychopharmacology, Swinburne University of Technology, Melbourne, VIC, Australia

**Keywords:** anxiety, working memory, language development, additional health and developmental needs, special health care needs children, special educational needs and disabilities

## Abstract

Although excessive childhood anxiety is recognised as a significant public health, education and socioeconomic concern, the specific effects of such anxiety on language development and working memory, particularly visual working memory, are relatively unknown. Thus, this study aimed to examine parent-reported trait anxiety, parent-reported functional language (daily communication skills) and clinical measures of non-verbal intelligence, receptive and expressive vocabulary, phonological awareness, and visual and auditory-verbal short-term and working memory in elementary schoolchildren. The final sample included 41 children categorised as Additional Health and Developmental Needs (AHDN) due to medical, neurodevelopmental or educational concerns and 41 age- and IQ-matched neurotypical (NT) children, aged 5- to 9-years. Results showed that 26% of all children in our entire sample (AHDN and NT) experienced moderate, sub-clinical anxiety (as reported by parents), and that AHDN children were 10.5 times more likely to experience high anxiety than the NT group (odds ratio). Parents of AHDN children reported lower functional language in their children than parents of NT children. Cognitive testing indicated that the AHDN group also had poorer visual and auditory-verbal working memory than the NT group. Further, High Anxiety children (drawn from both AHDN and NT groups) showed poorer parent-reported functional language skills, and lower visual and auditory-verbal working memory capacities. Our findings are amongst the first to confirm that the presence of high parent-rated trait anxiety is associated with reduced visual working memory in children, which is consistent with biological and theoretical expectations of the impact of anxiety on visually driven, goal-directed attention and working memory. Our results regarding the high prevalence of sub-clinical anxiety in both ADHD and neurotypical children highlight the need for early assessment of anxiety in all schoolchildren, especially those classified as AHDN.

## Introduction

Childhood anxiety is a significant public health concern for parents, psychologists and educational professionals worldwide (Creswell et al., 2020). The global prevalence of childhood anxiety disorders is 7% (Polanczyk et al., 2015; Goodsell et al., 2017), although in children categorised as Additional Health and Developmental Needs (AHDN), who experience chronic medical or neurodevelopmental conditions, the rates of anxiety reported are typically much higher (Manassis et al., 2007; Ghandour et al., 2010; van Steensel et al., 2011; Cross et al., 2019; Green et al., 2019). Further, early reports suggest that rates of anxiety symptoms in children and adolescents have doubled during the ongoing COVID-19 pandemic (Racine et al., 2021; Wang et al., 2022). This is particularly concerning, as high anxiety in childhood and adolescence has long been associated with a range of negative learning outcomes, including higher rates of absenteeism and school refusal, and poor academic performance (Hembree, 1988; Mazzone et al., 2007; Hadwin et al., 2016; Goodsell et al., 2017; Finning et al., 2019). However, the specific association between high childhood anxiety and other fundamental learning-related cognitive abilities, such as working memory or language, remains unclear. Thus, this study aimed to examine the effects of parent-reported trait anxiety on daily communication, language and working memory in elementary schoolchildren with both typical and atypical development.

Anxiety is a common human experience that can have physical, behavioural, and cognitive presentations (American Psychiatric Association, 2013; World Health Organization, 2018). Anxiety can be both adaptive and maladaptive, and is categorised into state anxiety, which refers to current and transient feelings of fear, worry or apprehension in response to a potentially threatening situation, and trait anxiety, which is a more enduring and maladaptive predisposition to experience worry or anxiety in a variety of contexts (Spielberger, 1972; Hadwin et al., 2016). Anxiety is often examined in the context of the broader categorisation of ‘emotional’ problems or ‘internalising’ symptoms, defined as a mixture of anxiety, mood and somatic symptoms (Achenbach, 1966; American Psychiatric Association, 2013). As noted above, AHDN children (who are also known as children with Special Health Care Needs [United States], or Special Educational Needs and Disabilities [United Kingdom]) are a group more likely to experience anxiety (Ghandour et al., 2010; Cross et al., 2019; Green et al., 2019). AHDN children are a heterogeneous group who “have, or are at increased risk (of), a chronic physical, developmental, behavioural or emotional condition and who also require health and related services of a type or amount beyond that required by children generally” (McPherson et al., 1998). Of note, AHDN children have been reported to commonly present with reduced speech, language and communication skills, as well as working memory difficulties (Astle et al., 2019; Gray et al., 2019; O'Connor et al., 2019; Siugzdaite et al., 2020). Whilst the heterogeneity of this population raises methodological challenges (Astle and Fletcher-Watson, 2020), AHDN children represent almost a quarter of children entering school each year (21–23%; O'Connor et al., 2019), and thus a more transdiagnostic approach to understanding this relatively high prevalence group is necessary (Astle et al., 2021).

Understanding of potential associations between childhood anxiety and language has grown substantially over recent years. Early reviews highlight that children with formally diagnosed language disorders (based on standardised testing) are almost twice as likely as their neurotypical peers to experience an internalising disorder in later childhood and adolescence (Yew and O'Kearney, 2013), and 81% of 5-13-year-olds experiencing ‘emotional or behavioural problems’ also have language difficulties (scores ≥1 SD below the mean on standardised testing; Hollo et al., 2014). More recently, higher anxiety symptoms (child or parent report) have been associated with lower language abilities on standardised testing, across childhood and adolescence (3–17-years; Hentges et al., 2021). Whilst these studies clearly suggest a link between language difficulties and broad emotional/internalising difficulties, what is not clear from previous work is whether there is a specific association between language and anxiety *per se*, or if another aspect of internalising symptoms (e.g. withdrawal, low mood) may be driving previous findings. Further, most previous studies have categorised language abilities in a binary fashion (i.e. impairment versus no impairment), rather than along a spectrum of language abilities (Hentges et al., 2021), and studies have rarely considered whether anxiety may impact any of the components of language differently (e.g. day-to-day functional communication, vocabulary knowledge or phonological processing). Thus, these are areas that require specific investigation.

Additionally, there is a wealth of adult literature suggesting that high state and trait anxiety primarily impacts working memory functioning (see Moran, 2016, for a comprehensive review). Working memory has traditionally been defined as a set of interrelated processes which include the simple storage of sensory information (i.e. short-term memory), and the more active manipulation of task-relevant information (i.e. working memory; Baddeley, 2012) within the current focus of attention (Cowan, 1999, 2017; Adams et al., 2018). Theoretically, the primary cognitive impact of high anxiety should be seen on visual working memory (including both spatial and visuo-perceptual information), given that anxiety is thought to impair goal-directed attentional control within working memory (Eysenck and Derakshan, 2011), which like attention is neuroanatomically known to be predominately visually driven (Corbetta and Shulman, 2002; D'Esposito and Postle, 2015). Whilst this is largely the case in adult studies (Eysenck et al., 2005; Shackman et al., 2006; Moran, 2016), results from child studies are not as clear. Indeed, the small number of child studies conducted to date have linked both high state and trait anxiety with poorer verbal working memory (Hadwin et al., 2005; Owens et al., 2008; Visu-Petra et al., 2009, 2011, 2014; Ng and Lee, 2010, 2015, 2016), but not with spatial working memory (Hadwin et al., 2005; Owens et al., 2008, 2014; Visu-Petra et al., 2011, 2014, 2018), or visuo-perceptual working memory (e.g. colour memory; Toren et al., 2000; Visu-Petra et al., 2011). Thus, better understanding of how anxiety may impact working memory in children, particularly relatively understudied visuo-perceptual memory, is an area that requires further investigation.

Hence, the current study aimed to investigate the association between parent-report of trait anxiety, parent-perception of functional language abilities, several specific language components (receptive and expressive vocabulary, phonological awareness) and visual and auditory-verbal working memory, in a sample of AHDN children and their neurotypical (NT) peers. Specifically, our aims were to:

1. Assess the relative incidence of parent-reported trait anxiety in AHDN and NT children, to investigate if parent-reported anxiety is more common in ADHD or NT children. We hypothesised that clinical and sub-clinical levels of trait anxiety would be more common in AHDN children compared to NT children.
2. Compare AHDN and NT children on parent-perceived functional language, performances on standardised vocabulary and phonological awareness measures and working memory abilities. We hypothesised that AHDN children would perform less well than their NT peers on all language and cognitive measures.
3. Investigate how language and working memory abilities are related to parent report of anxiety, hypothesising that anxiety would correlate negatively with measures of working memory and language.
4. Compare children high and low in parent-reported trait anxiety on language and working memory performances. We hypothesised that children with high parent-reported anxiety would also have lower parent-perceived functional language skills, and have lower language and working memory performances, than children with low parent-reported anxiety.

## Materials and methods

### Participants

Participants were recruited from four different locations in metropolitan Melbourne, Australia, between May 2018 and January 2020 (prior to the COVID-19 Pandemic in Australia) including three mainstream schools and one holiday program for mainstream schoolchildren experiencing school-based problems with language, academic subjects or social–emotional functioning. A previous study in this area (Cross et al., 2019) also collected data from the same Holiday program, however, this occurred before our study commenced, and thus there was no overlap in participants.

Study inclusion criteria were the same for all recruitment locations: children were required to be aged 5- to 9-years old, with normal or corrected-to-normal hearing and vision, and to speak English as their primary language. This research was conducted in accordance with the Declaration of Helsinki, and ethical approval was granted by all relevant university and education committees.

Two groups were created, matched on age (within 5 months, *M* = 1.5 months, *SD* = 1.2 months) and non-verbal intelligence (within seven IQ points, *M* = 2.37, *SD* = 1.83). The AHDN group consisted of 27 children recruited from the holiday program and 14 children recruited from the mainstream schools (*n* = 41). To be included in the AHDN group, children had to meet the above study inclusion criteria, and at least one of the following: (1) parent report of a diagnosed neurodevelopmental disorder, (2) parent report that the child was currently undergoing clinical assessment for cognitive, learning or behavioural difficulties, (3) parent and teacher report of school-based language, academic or social–emotional difficulties or (4) a standard score at or below 85 on a literacy screen (see *Measures* below). Of the 41 AHDN children, 18 (44%) had a formal diagnosis of a neurodevelopmental disorder, 1 (2%) was undergoing assessment and 22 (54%) had no formal diagnosis (but had concerned parents and teachers). Six of these undiagnosed children (27%) also failed the literacy screen.

The NT sample consisted of 41 age- and IQ-matched children drawn from a larger sample of 80 mainstream schoolchildren who met the above study inclusion criteria, scored above 85 on the literacy screen (see *Measures* below) and whose parents reported they had not been diagnosed with, nor were currently undergoing assessment for, any neurodevelopmental disorders. Descriptive information in presented in Table 1. A G*Power analysis (Faul et al., 2007) indicated that two groups of 41 children had adequate power (70–80%) to detect large effect sizes, but was underpowered to detect medium or small effect sizes. However, local restrictions due to the COVID-19 Pandemic precluded further data collection.

**Table 1:** Descriptive information and Mann–Whitney U test results comparing matched AHDN and NT groups.

	AHDN (*n* = 41)		NT (*n* = 41)		*p*	Effect size, *r*
	*M* (*SD*)	Range	*M* (*SD*)	Range		
Age	7.06 (1.06)	5.05–9.05	7.04 (1.02)	5.28–9.27	0.948	0.01
RCPM SS	108.78 (11.84)	89–139	109.10 (10.85)	92–133	0.878	0.02
Total SCAS-P	19.18 (14.01)	1–61	12.72 (8.48)	0–34	0.038	0.23
Vis DS-F	3.63 (1.04)	2–6	4.41 (1.10)	2–7	0.002	0.34
Aud DS-F	4.49 (1.00)	2–7	4.93 (1.06)	3–8	0.035	0.23
Vis DS-B	2.56 (1.11)	0–5	3.29 (1.06)	0–5	0.003	0.32
Aud DS-B	2.44 (0.92)	0–4	3.20 (0.72)	2–5	<0.001	0.43
PPVT SS	105.56 (11.76)	89–129	113.24 (10.60)	87–131	0.003	0.32
EVT SS	97.17 (9.67)	76–117	110.54 (11.43)	87–131	<0.001	0.54
Elision SS	7.98 (1.86)	3–12	10.90 (2.29)	7–16	<0.001	0.57
ALDeQ-Early	0.86 (0.19)	0.33–1.00	0.95 (0.12)	0.33–1.00	0.004	0.32
ALDeQ-Current	0.62 (0.25)	0.07–1.00	0.89 (0.09)	0.67–1.00	<0.001	0.55

AHDN, additional health and developmental needs; NT, neurotypical; RCPM, Raven’s coloured progressive matrices; SS, standard score (*M* = 100, *SD* = 15); SCAS-P, spence children’s anxiety scale – parent; Vis, visual; DS-F, digit span forward; Aud, auditory; DS-B, digit span backward; PPVT, Peabody picture vocabulary test; EVT, expressive vocabulary test; ALDeQ, Alberta language and development questionnaire.

### Measures

#### Literacy screening

Literacy screening for participants from mainstream schools was conducted to assist with grouping participants, using the York Assessment of Reading for Comprehension (YARC). Early readers (Grades Prep/Foundation, One and Two) completed the Early Reading Letter-Sound Knowledge Test (YARC-LSK; Hulme et al., 2012) and older children (Grades Three and Four) completed the Primary School Passage Reading task (YARC-PR; Snowling et al., 2012). In the YARC-LSK, children are presented with a single page and asked to provide the appropriate sound for all 26 letters (randomised order) and 5 diagraphs (“th,” “sh,” “ch,” “oo,” and “ee”), as per the standardised instructions. Raw scores were converted to standard scores according to the manual’s norms for children aged between 5;0 and 7;11. For seven children who completed the YARC-LSK but were above 7;11 (aged 8;0–8;4), their results were scored using the 7;11 norms. The manual reports strong reliability within the normative sample (*α =* 0.91; Hulme et al., 2012). In the YARC-PR, children are required to read two short passages aloud to the examiner, who records errors and reading speed. Reading accuracy on the YARC-PR was used as the primary score as it is most comparable to the YARC-LSK task the younger participants completed. Reported reliability is moderate (*α* = 0.63–0.77; Snowling et al., 2012).

#### Intellectual functioning

Non-verbal intelligence was assessed with the Raven’s Coloured Progressive Matrices test (RCPM; Raven et al., 1998) as it is a quick, engaging and reliable (*α* = 0.81–0.91) measure of non-verbal intelligence that has been validated in Australian schoolchildren (Cotton et al., 2005). The RCPM contains 36 different items of varying complexity. Each item is an incomplete, coloured matrix that the participant is asked to complete by selecting one of six alternate options. Standard scores were calculated based on chronological age (in years), using normative data from Cotton et al. (2005) for children aged 6-years and above, and from Raven et al. (1982) for children aged 5-years.

#### Anxiety

Parent-reported trait anxiety was measured using the Spence Children’s Anxiety Scale, Parent Report (SCAS-P; Nauta et al., 2004), which requires parents to rate 38 anxiety-related behaviours on a Likert scale (never [0], sometimes [1], often [2], always [3]). The SCAS-P measures generalised anxiety (e.g. “my child worries about things”), specific fears/phobias (e.g. “my child is scared of the dark”), separation anxiety (e.g. “my child worries about being away from us/me”), social anxiety (e.g. “my child worries what other people think of him/her”), panic and agoraphobia (e.g. “my child is afraid of being in closed places, like tunnels or small rooms”) and obsessive–compulsive behaviours (e.g. “my child has to do some things over and over again like washing his/her hands, cleaning or putting things in a certain order”). The Total score (all responses summed) was used in this study. Parent report was chosen over child-report due to the age of our youngest participants (5–6-years), who may not be able to reliably self-report on trait anxiety. The SCAS-P has been validated in Australian children with and without anxiety disorders and reports strong reliability (*α* = 0.89; Nauta et al., 2004).

#### Short-term and working memory

Short-term and working memory were assessed with the common neuropsychological test Digit Span (Lezak et al., 2012; Wechsler, 2016) in two modalities: auditory and visual. We note that whilst a visual presentation of digits requires the participant to initially process the information visually, they may then support or supplement the visual information with silent verbal rehearsal, meaning they may use both visual short-term/working memory alone or a more multimodal approach. Such a multisensory approach is likely only applicable in older children (from 7-years), as previous literature suggests that younger children typically do not use verbal rehearsal strategies (Hitch et al., 1988; Pickering, 2001), and younger children were potentially less familiar with the visual forms of digits. Whilst this may partially confound our ability to interpret the visual Digit Span task as a wholly visual measure, variation of presentation modality allowed us to assess short-term and working memory with the same task in both modalities, in order to directly compare the potential impact of anxiety on information processing through both senses.

In the Digit Span task, children were presented with a sequence of digits auditorily (read aloud by the researcher) or visually (on a computer screen) at a rate of one digit per second. Children responded verbally in the auditory condition, however, in the visual condition children could choose to respond verbally or to point to the digits on a keypad presented on the screen during the response phase. Whilst response method data was not collected, the majority of children chose to respond verbally. All children completed both versions of the task in a counterbalanced order. In the Forward condition (DS-F), indexing short-term memory, children were required to repeat the sequence in the same order as presented, and in the Backward condition (DS-B), indexing working memory, children were required to repeat the sequence in reverse order. The task begins with a sequence length of two digits, presents two trials at each sequence length and is discontinued when both sequences of a particular length are incorrect.

Reliability estimates for auditory Digit Span are moderate to good in neurotypical children (0.75–0.85; Cohen, 1997; Wechsler, 2016), and in atypical populations (including learning disorders, Autism Spectrum Disorders and Attention-Deficit/Hyperactivity Disorder; Forward *M* = 0.86; Backward *M* = 0.88; Wechsler, 2016). The custom visual digit span task has previously been used with a range of typical and atypical children (e.g. Mungkhetklang et al., 2016; Alghamdi et al., 2021) and may be considered a more reliable estimate of working memory (than auditory-verbal digit span) in populations with language difficulties (Olsthoorn et al., 2014). Reliability in our sample was moderate to good in both the NT (Forward = 0.70; Backward = 0.82) and AHDN groups (Forward = 0.71; Backward = 0.84).

#### Language measures

##### Vocabulary

Receptive vocabulary was assessed with the Peabody Picture Vocabulary Test – Fourth Edition (PPVT-IV; Dunn and Dunn, 2007). Children were presented with an array of four pictures and asked to select the picture that matched an orally presented word. Expressive vocabulary was measured with the Expressive Vocabulary Test – Second Edition (EVT-2; Williams, 2007). Children were asked to provide a short verbal description or a synonym for a presented picture. Administration and scoring of the PPVT and EVT was conducted according to the manualised instructions. Both the PPVT and EVT report strong reliability estimates within the normative samples (PPVT *α* = 0.95–0.97, Dunn and Dunn, 2007; EVT *α* = 0.94–0.96, Williams, 2007).

##### Phonological awareness

Phonological awareness was measured with the Elision subtest from the Comprehensive Test of Phonological Processing, Second Edition (CTOPP-2; Wagner et al., 2013). Children were first asked to repeat an orally presented word (e.g. “say popcorn” or “say bold”) and then asked to repeat the word without a particular element (e.g. “say popcorn without saying/corn/” or “say bold without saying /b/”). Reliability in the normative sample is strong (*α* = 0.91–0.93; Wagner et al., 2013).

##### Parent report of language

Parents reported on their child’s language abilities using an adapted version of the Alberta Language and Development Questionnaire (ALDeQ; Paradis et al., 2010). The ALDeQ was designed to be a comprehensive assessment of language development and current language skills that is not specific to any one language or culture. In the current study, we used the language development questions from the first section (age of first spoken word and age when first combining words; ALDeQ-Early), and current functional language abilities from the second section (e.g. how well your child express themselves, how easy is it to have a conversation with your child; ALDeQ-Current). The specific questions used and scoring procedure are provided in the Supplementary material. Scores in each section are averaged, with possible scores ranging between 0 (indicating very poor language development) to 1 (indicating strong language development). In the normative sample (Paradis et al., 2010), the average overall score for early language milestones was 0.88 for neurotypical children and 0.45 for children with language impairments, and the average score for current language abilities was 0.73 in neurotypical children and 0.38 for children with language impairments. Reliability data for the normative sample were not reported (Paradis et al., 2010). In our sample, reliability for ALDeQ-Early was moderate in NT children (*α* = 0.62) and low in AHDN children (*α* = 0.36), and reliability for the ALDeQ-Current was moderate in NT children and (*α* = 0.62) and strong in AHDN children (*α* = 0.92).

### Procedure

Children were seen for assessment after signed ethics consent forms had been returned, which included the completed SCAS-P and ALDeQ questionnaires. Verbal assent was obtained by children at the start of each assessment session, and children were informed that they could request to discontinue at any point of the session. Children received a sticker or stationary item (i.e. pencil, eraser) as a thank you at the end of each session.

Assessments were conducted over 3–4 sessions of up to 30-min, either in the same week (Holiday Program) or over the course of 2–3 weeks (Schools), including some additional tasks not reported in the current study. Session number and length varied depending on engagement and attention of the child, equipment availability and external scheduling factors (e.g. meal breaks and activities). The order of the auditory and visual Digit Span tasks was counterbalanced, with the two versions completed in different sessions. Otherwise, there was no set task order. Tasks were administrated by psychology or speech pathology students who were trained in standardised administration and supervised by qualified and experienced Speech Pathologists or Psychologists.

### Data screening and analysis

Data for the AHDN and NT groups were screened separately, with no univariate (z-scores > ± 3.29) or multivariate (Cook’s Distance >1) outliers detected. However, assumption testing (Kolmogorov–Smirnov, Levene’s Test) indicated several variables (Digit Span, Elision, PPVT, SCAS-P, ALDeQ) were not normally distributed in at least one group, and homogeneity of variance was violated for Elision, SCAS-P and ALDeQ. Hence, non-parametric statistics were used for analyses.

Data were analysed using IBM SPSS 27.0. To account for multiple comparisons whilst maintaining sufficient statistical power, the criterion for significance was set to <0.01, and effect sizes were used to aid interpretation of results (Rothman, 1990; Cumming, 2011). First, a Chi-square analysis and an odds ratio were used to compare the relative incidence of anxiety in the two groups (AHDN and NT). This was achieved by comparing Total SCAS-P scores for the two groups against the published normative data from Nauta et al. (2004). Scores at or below the mean for a non-anxious child (raw score ≤ 16) were considered to represent low anxiety, and scores at or above the mean for an anxious child (raw score ≥ 31 for males and ≥ 33 for females) were considered to represent high anxiety. Scores that fell in between this (17–30 for males and 17–32 for females) were considered to represent potential sub-clinical anxiety.

Next, the Mann–Whitney U-test was used to compare the two groups on all cognitive, language and anxiety measures. Partial correlations (controlling for age) were then used to examine the association between cognitive, language and anxiety measures across the entire sample. Finally, to examine the impact of anxiety on working memory and language, a tertile split was used to compare children with High Anxiety (top third) to those with Low Anxiety (bottom third) with the Mann–Whitney U-test.

## Results

### Relative incidence of anxiety in AHDN and NT groups

The number of children reported to have low, sub-clinical or high anxiety is reported in Table 2. The relative incidence of anxiety between the two groups (AHDN and NT) was compared using a 2 × 3 Chi-square analysis, which found a nonsignificant association between group and level of anxiety (*χ*^2^ (2) = 6.41, *p* = 0.041), with a small effect size (Cramer’s *V* = 0.28). These results suggest that children with AHDN are more likely to experience High anxiety (19.5% vs. 2.4% of NT children) than Low/Normal anxiety (53.6% vs. 70.7% of NT children). Sub-clinical anxiety was equal between the groups (26.8%). When comparing the proportions of Low/Normal and High anxiety between the two groups (odds ratio), children with AHDN were 10.5 times more likely to have High anxiety (compared to Low/Normal anxiety) than NT children.

**Table 2:** Number and percentage of children with parent-reported low/normal, sub-clinical and high anxiety.

	AHDN	NT	Total
Low/normal	22 (53.6%)	29 (70.7%)	51 (62.2%)
Sub-clinical	11 (26.8%)	11 (26.8%)	22 (26.8%)
High	8 (19.5%)	1 (2.4%)	9 (11%)

AHDN, additional health and developmental needs; NT, neurotypical.

### Comparing AHDN and NT children on working memory and language performances

Descriptive statistics and results of the Mann–Whitney U tests comparing the matched AHDN and NT participants on all variables are presented in Table 1. The AHDN group performed less well than the NT group on all language measures, with medium to large effect sizes (*r* = 0.32–0.57). Of note, whilst the group differences on both vocabulary measures (PPVT and EVT) were significant, these differences are likely not clinically meaningful as the means for the AHDN group were still within the typical range (standard score of 90–110). Whilst parent-reported anxiety levels were greater for the AHDN group (*M* = 19.18) than for the NT group (*M* = 12.72), this difference did not meet statistical significance at the corrected level and showed only a small effect size (*r* = 0.23). Additionally, the AHDN group performed significantly worse than the NT group on most short-term and working memory measures (all expect auditory DS-F), with medium effect sizes (*r* = 0.34–0.43).

### Associations between anxiety, working memory and language

Partial correlations (controlling for age) for the entire sample are provided in Table 3. Parent-reported anxiety scores had a significant, small, negative correlation with parent report of current language abilities (*r* = −0.297, *p* = 0.007). Additionally, parent-reported anxiety scores showed a significant, small, negative correlation with visual working memory (Visual DS-B *r* = −0.290, *p* = 0.009), and although a similar correlation with auditory working memory was found, this did not reach significance at the corrected level (Auditory DS-B *r* = 0.273, *p* = 0.014). A similar pattern of results was also found when examining the AHDN and NT groups separately (see the Supplementary material; Supplementary Table S2).

**Table 3:** Partial correlations (and 95% CI) between all measures.

	RCPM	SCAS-P	Vis DS-F	Aud DS-F	Vis DS-B	Aud DS-B	PPVT	EVT	Elision	ALDeQ-E	ALDeQ-C
RCPM	-										
SCAS-P	0.028 (−0.194, 0.247)	-									
Vis DS-F	0.001 (−0.220, 0.222)	−0.094 (−0.309, 0.130)	-								
Aud DS-F	0.148 (−0.076, 0.357)	0.069 (−0.155, 0.286)	0.487*** (0.298, 0.639)	-							
Vis DS-B	0.314** (0.100, 0.500)	−0.290** (−0.480, −0.074)	0.379*** (0.172, 0.554)	0.310** (0.095, 0.497)	-						
Aud DS-B	0.296** (0.080, 0.485)	−0.273* (−0.466, −0.055)	0.431*** (0.232, 0.595)	0.202 (−0.020, 0.405)	0.598*** (0.434, 0.724)	-					
PPVT	0.382** (0.176, 0.556)	−0.144 (−0.354, 0.080)	0.431*** (0.232, 0.595)	0.362*** (0.153, 0.540)	0.290** (0.074, 0.480)	0.390*** (0.185, 0.563)	-				
EVT	0.186 (−0.036, 0.391)	−0.168 (−0.375, 0.055)	0.365*** (0.157, 0.542)	0.129 (−0.095, 0.340)	0.434*** (0.236, 0.598)	0.403*** (0.200, 0.573)	0.573*** (0.403, 0.705)	-			
Elision	0.404*** (0.200, 0.574)	−0.232* (−0.431, −0.012)	0.474*** (0.283, 0.629)	0.336** (0.124, 0.519)	0.582*** (0.414, 0.712)	0.498*** (0.312, 0.648)	0.402*** (0.199, 0.572)	0.544*** (0.367, 0.683)	-		
ALDeQ-E	0.070 (−0.153, 0.287)	−0.193 (−0.397, 0.029)	0.271* (0.053, 0.464)	0.203 (−0.019, 0.406)	0.246* (0.026, 0.443)	0.399*** (0.195, 0.570)	0.229* (0.008, 0.428)	0.202 (−0.020, 0.405)	0.247* (0.027, 0.444)	-	
ALDeQ-C	−0.155 (−0.364, 0.069)	−0.297** (−0.486, −0.081)	0.361*** (0.152, 0.539)	0.227* (0.006, 0.426)	0.346** (0.135, 0.527)	0.464*** (0.270, 0.621)	0.387*** (0.181, 0.560)	0.515*** (0.332, 0.661)	0.397*** (0.193, 0.568)	0.374*** (0.167, 0.550)	-

Partial correlation controlling for age. *N* = 82, *df* = 79. RCPM, Raven’s coloured progressive matrices; SCAS-P, spence children’s anxiety scale – parent; Vis, visual; DS-F, digit span forward; Aud, auditory; DS-B, digit span backward; PPVT, Peabody picture vocabulary test; EVT, expressive vocabulary test; ALDeQ, Alberta language and development questionnaire; E, early; C, current. *p ≤ 0.05, **p ≤ 0.01, ***p ≤ 0.001.

### Comparing low and high anxiety children

To examine how anxiety may impact language and working memory abilities transdiagnostically, children with low and high parent-reported anxiety were compared, using a tertile split to find the bottom and top third of children (based on anxiety ratings). This resulted in a Low Anxiety group (*n* = 26) where 35% were children from the AHDN group and 65% were children from the NT group. The High Anxiety group (*n* = 28) was comprised of 64% AHDN children and 36% NT children. Although these smaller group sizes further reduce statistical power, such analyses are nevertheless important for exploring the specific effect of anxiety on working memory and language abilities.

Results of the Mann–Whitney U tests comparing the Low and High anxiety groups are presented in Table 4. The only significant difference observed is for visual working memory (Vis DS-B), where the visual working memory span of High Anxiety children is lower than Low Anxiety children (medium effect size, *r* = 0.37). There are also differences between the groups for auditory working memory (Aud DS-B) and parent report of current language (ALDeQ-Current), both with a medium effect size (*r* = 0.32 and 0.33, respectively); however, these differences did not meet statistical significance at the corrected level.

**Table 4:** Descriptive information and Mann–Whitney U test results low and high anxiety groups.

	Low anxiety (*n* = 26)		High anxiety (*n* = 28)		*p*	Effect size, *r*
	*M* (*SD*)	Range	*M* (*SD*)	Range		
Age	7.20 (1.11)	5.28–9.27	6.98 (1.11)	5.37–9.06	0.516	0.09
RCPM SS	107.81 (11.16)	91–131	108.18 (10.45)	89–130	0.742	0.04
Total SCAS-P	5.02 (2.52)	0–8.5	29.03 (10.73)	19–61	<0.001	0.86
Vis DS-F	4.47 (1.15)	2–7	3.86 (1.08)	2–6	0.190	0.18
Aud DS-F	4.73 (0.83)	2–6	4.71 (1.15)	2–7	0.767	0.04
Vis DS-B	3.50 (0.91)	2–5	2.54 (1.31)	0–5	0.006	0.37
Aud DS-B	3.19 (0.63)	2–5	2.54 (1.11)	0–4	0.017	0.32
PPVT SS	108.58 (10.77)	89–131	107.79 (12.98)	89–130	0.345	0.03
EVT SS	105.77 (13.17)	89–131	100.39 (12.30)	76–120	0.222	0.17
Elision SS	10.19 (2.68)	4–16	9.00 (2.33)	5–14	0.102	0.22
ALDeQ-Early	0.95 (0.09)	0.67–1.00	0.86 (0.21)	0.33–1.00	0.123	0.21
ALDeQ-Current	0.81 (0.03)	0.13–1.00	0.68 (0.23)	0.07–1.00	0.015	0.33

AHDN, additional health and developmental needs; NT, neurotypical; RCPM, Raven’s coloured progressive matrices; SS, standard score (*M* = 100, *SD* = 15); SCAS-P, spence children’s anxiety scale – parent; Vis, visual; DS-F, digit span forward; Aud, auditory; DS-B, digit span backward; PPVT, Peabody picture vocabulary test; EVT, expressive vocabulary test; ALDeQ, Alberta language and development questionnaire.

## Discussion

This study utilised a transdiagnostic approach to examine the effects of parent-perceived trait anxiety on working memory and language abilities in a mixed sample of age- and IQ-matched AHDN and NT children. We found that AHDN children were more likely to experience high anxiety than their NT peers, and that the AHDN group performed worse than the NT group on all working memory and language measures. More importantly, a quarter of the entire sample (26.8%) was reported by parents to experience moderate, sub-clinical trait anxiety and children who experienced high anxiety had reduced working memory performances and lower parent-reported functional language skills, regardless of original group membership (AHDN or NT).

### Comparing AHDN and NT children

In contrast to our predictions, group comparisons revealed only a small difference between AHDN and NT children on parent-reported anxiety; however, analysis of anxiety severity revealed that 19% of AHDN children experienced high anxiety (at or above the mean of an anxiety-disordered child), compared to just 2% of NT children. These findings are similar to previous studies, where Ghandour et al. (2010) found 16% of 3–5-year-old AHDN children experienced ‘internalising’ symptoms (inclusive of, but not limited to, anxiety), and Green et al. (2019) found 16% of 6–11-year-old AHDN children experienced anxiety specifically. Indeed, in our sample, an odds ratio indicated that AHDN children were 10.5 times more likely than NT children to experience high anxiety (compared to low anxiety). Our results also revealed that both AHDN and NT children were equally likely to experience sub-clinical anxiety, with just over a quarter (26.8%) of all children in our sample reported by parents as being moderately anxious. These results highlight the importance of rigorous assessment of anxiety in all schoolchildren, particularly in the wake of the COVID-19 Pandemic, during which the prevalence of anxiety in children and adolescents has increased significantly (Racine et al., 2021; Wang et al., 2022). How this applies to AHDN children in particular, who were already at a higher risk of experiencing anxiety, awaits further investigation.

Additionally, as predicted, AHDN children had poorer short-term and working memory abilities than NT children, which is consistent with previous research in broad AHDN samples (children referred for school-based difficulties; Astle et al., 2019), and other neurodevelopmental conditions (Schuchardt et al., 2008; Hutchinson et al., 2012; Kasper et al., 2012; Habib et al., 2019). This finding suggests that working memory deficits are a common experience in AHDN children, irrespective of specific diagnosis (Astle et al., 2019; Gray et al., 2019). Regarding language, parents of the AHDN children perceived their children’s current functional language abilities to be much below that of their peers (large effect size). Although a small portion of our AHDN sample (*n* = 6, 15%) had diagnosed speech or language disorders, the pattern and strength of these findings did not change when removing these 6 children (and their matched NT pairs) from the analysis (see the Supplementary material; Supplementary Table S3). Indeed, past research has found that up to 22% of AHDN children have parent-reported speech and/or language difficulties (O'Connor et al., 2016), and as many as 58% of AHDN children have teacher-reported speech and/or language difficulties (O'Connor et al., 2019). Together, this suggests that language difficulties are likely to be a common occurrence amongst AHDN children, even in the absence of a clinically defined formal language disorder.

What is less clear from our findings is why or how nearly 20% of our AHDN sample experience higher anxiety, and lower language and working memory. Possibly, AHDN children may be genetically more susceptible to experience anxiety, or the nature of their additional needs may make them more likely to encounter adverse life experience (e.g. social difficulties) that contribute to anxiety symptoms. Furthermore, comorbidly impaired language and working memory are also likely to affect and even limit social and academic functioning, which in turn would be expected to increase anxiety, particularly in the school context (Young et al., 2002; Gathercole et al., 2004; Astle et al., 2019; Finning et al., 2019; Ziegenfusz et al., 2022); indeed, it is known that AHDN children are at greater risk of poor school outcomes (Bethell et al., 2012). However, our results also suggest that anxiety is not a parent-perceived experience for all AHDN children, given 53% of our AHDN sample display low/normal levels of anxiety, and similarly there were some AHDN children who had stronger and more neurotypical working memory and language performances. Future longitudinal studies tracking the development of AHDN children will be beneficial to better understand these associations, and the potential underlying mechanisms.

### The effect of anxiety

Our final two aims were concerned with the potential impact of anxiety on working memory functioning and language abilities. First, correlational analyses in the whole sample (partial correlations controlling for age, given the large age range of our participants) found, as predicted, that parent-reported trait anxiety was negatively correlated with both visual and auditory-verbal working memory, although only visual working memory was significant at our corrected threshold. Regarding language, the only significant correlation was with parent report of current language abilities, suggesting higher anxiety is associated with lower functional language and communication skills. When comparing children high and low in anxiety (top and bottom third of the combined sample), results followed a similar pattern, with high anxiety children having a significantly lower visual working memory capacity than low anxiety children (medium effect size). There were similar differences for auditory working memory and parent report of current language (both medium effect sizes), however, these did not reach significance at the corrected level.

These findings demonstrate the association between anxiety and language abilities, showing that parents who rate their children as more anxious also perceive their children as having difficulty using language for functional communication in day-to-day settings. This is consistent with a recent study by Sbicigo et al. (2020), who found that more severe anxiety symptoms were associated with overall lower oral language abilities, in children with anxiety disorders. Although our high anxiety group contained a higher proportion of AHDN children, none of these children had a formal diagnosis of a clinical anxiety disorder, nor did the one-third of the high anxiety group who were NT children, indicating that anxiety may impact language abilities at a level below formal diagnosis of an anxiety disorder. Indeed, a recent meta-analysis by Hentges et al. (2021) found a small association between ‘internalising’ symptoms (inclusive of, but not limited to, anxiety) and language abilities both when language was considered categorically (impairment versus no impairment), and on a dimensional scale, highlighting that anxiety may also impact language outside of a formal language disorder. As much of the previous research examining associations between language and psychopathology has broadly considered ‘internalising’ or ‘emotional’ symptoms (e.g. combined measures of mood, anxiety and somatic symptoms; Yew and O'Kearney, 2013; Hollo et al., 2014; Helland et al., 2018), our results extend this past work by demonstrating that specifically anxiety, rather than other internalising symptoms such as low mood, is related to language difficulties.

Additionally, our results are consistent with a large body of literature in adults, and a smaller number of studies in children, showing that trait anxiety is associated with poorer working memory performance for auditory and spatial information (see Moran, 2016, for a review). To our knowledge, this study is amongst the first to show that anxiety is related to performances on visual working memory tasks that require minimal spatial processing, as previous research in children has largely focused only on spatial working memory, and mostly found no significant association with anxiety (Hadwin et al., 2005; Owens et al., 2008, 2014; Visu-Petra et al., 2011, 2014; although see Visu-Petra et al., 2018; Sbicigo et al., 2020). Further, the few previous studies considering non-spatial, visuo-perceptual working memory (i.e. highly salient visuo-perceptual details, such as colours) have also found no significant associations (Toren et al., 2000; Visu-Petra et al., 2011). In contrast, our findings on a more complex temporal visuo-perceptual working memory task are consistent with biological and theoretical expectations, given that neuroanatomical brain imaging demonstrates that goal-directed attention is primarily visually driven (Corbetta and Shulman, 2002; D'Esposito and Postle, 2015). As anxiety has been proposed to impact working memory via disruption of goal-directed attention (Eysenck and Derakshan, 2011), our findings also lend behavioural support to suggestions that similar parieto-frontal neural networks are involved in the biological and cognitive response to stress and anxiety (Godoy et al., 2018; Lamotte et al., 2021). However, more research to specifically pinpoint the underlying mechanisms of how anxiety effects working memory is clearly needed. In addition, to our knowledge, this is the first study to consider visual digit span (alongside traditional auditory-verbal digit span) in relation to anxiety. Whilst our findings require replication, they emphasise the importance of assessing all aspects of visual working memory (Pickering et al., 2022), using comparable visual and auditory/verbal tasks, to allow for a clearer comparison between the two sensory modalities (Shackman et al., 2006; Visu-Petra et al., 2006).

Furthermore, whilst our findings demonstrate that AHDN children are much more likely to experience high anxiety than their NT peers, experiencing anxiety is not unique to the AHDN group. This is evident from the 26% of NT children who experience moderate/sub-clinical anxiety, and the fact that 36% of our High Anxiety group (the top third of anxiety scores across both groups) were NT children. Importantly, regardless of whether a child was classified as AHDN or NT, experiencing high anxiety was associated with poorer visual and auditory-verbal working memory, and reduced parent-reported functional language abilities. This further underscores the importance of assessing anxiety in all children, especially as child circumstances change (i.e. as a result of the COVID-19 Pandemic), and highlights the importance of a dimensional, transdiagnostic approach to psychopathology and neurodevelopmental research (Dalgleish et al., 2020; Astle et al., 2021).

### Limitations and future directions

The primary limitation of this study is the small sample size and limited statistical power. Although G*Power analyses suggested good statistical power for large effect sizes (Faul et al., 2007), there may be smaller, subtler differences unable to be detected within our study, particularly regarding comparisons between Low and High anxiety children, where group sizes were smaller. Additionally, the inclusion of a wide range of children within the AHDN sample makes it difficult to generalise our findings to particular subsets of children who fall within this group. However, the use of a heterogeneous group was also an important strength of this study, as it allowed us to examine the associations between anxiety, language and working memory without the constraints of traditional clinical diagnostic boundaries (Astle et al., 2021). Whilst our results require replication in larger samples to be more clearly interpreted, they nevertheless provide useful guidance for future research in this area, particularly regarding the importance of anxiety assessment for all children, and the need to examine working memory in different sensory modalities (e.g. Pickering et al., 2022).

It is also important to acknowledge that our visual Digit Span task may not be considered a ‘pure’ measure of visual working memory; whilst information was initially presented to children visually, children may have internally supplemented the visual presentation with auditory rehearsal of the verbalisable information, and how each child stored the visually acquired information within working memory is unknown. Our youngest participants had completed at least 4-months of formal schooling, thus ensuring that they were at least somewhat familiar with the semantic labels of the visually presented digits, making visual or verbal encoding strategies possible. However, in our younger children (i.e. 7-years and under) who are still learning to read, verbal encoding and rehearsal strategies are reported to be unlikely (Hitch et al., 1988; Pickering, 2001; Alghamdi et al., 2021). It is therefore possible that some of the association between visually presented Digit Span and anxiety may be mediated by verbal encoding and rehearsal factors, although other aspects of our results suggest that the impact of such verbal encoding is small: for instance, the correlations between auditory-verbal and visual forward digit span (short-term memory) and backward digit span (working memory) are only moderate (0.49 for forwards and 0.59 for backwards), indicating that the two task modalities are at least partially differentiated. Further, mean scores and ranges do differ (primarily on digit span forward), again suggesting differentiation in the processing of visual and auditory-verbal presentations of digits. Thus, whilst we may be unable to draw firm conclusions regarding the impact of anxiety on visual working memory *per se*, we can comment on performance differences in processing information presented via different modalities, with our results suggesting that anxiety impacts the processing of both auditory-verbal and visually presented information. Future research including a variety of visual memory tasks would be beneficial to better understand how anxiety may impact visual working memory.

Finally, our findings regarding anxiety are limited to only parent report of trait anxiety. Whilst parental perception of child anxiety is reported to be a valid and reliable measure of trait anxiety (Nauta et al., 2004; Whiteside and Brown, 2008; Orgilés et al., 2019), and was more appropriate than self-report for our youngest participants (5–6-years), it has also been recognised that parent and child reports can reflect different aspects of anxiety (Cole et al., 2000), and so it will be beneficial for future research to consider both parent and child reports of anxiety. Further, we did not include an objective measure of state anxiety, and as it is presently unclear from the existing literature how this may interact with trait anxiety to impact cognitive performance (Ng and Lee, 2015; Moran, 2016; Ng and Lee, 2016), more research is needed in this area. It may also be useful for future research to include teacher reports of classroom anxiety levels (e.g. Aronen et al., 2005), to better examine the applicability of our findings to the classroom setting, where most AHDN children experience difficulties (Bethell et al., 2012; Goldfeld et al., 2015).

### Conclusion

High childhood anxiety is a significant public health concern, given the long-term negative social, emotional and educational consequences, and rising prevalence during the COVID-19 Pandemic (Hadwin et al., 2016; Racine et al., 2021; Wang et al., 2022). The current study demonstrated that AHDN children are 10.5 times more likely to experience high anxiety than their NT peers, and that a quarter of all children experience moderate, sub-clinical anxiety. Importantly, irrespective of their original group (AHDN or NT), children with high anxiety had lower visual and auditory-verbal working memory capacities, and poorer parent-rated functional language skills. This highlights the negative effects of anxiety on cognitive functioning, and thus the importance of considering anxiety levels in all children. For AHDN children, who are at a significantly higher risk of experiencing clinically elevated anxiety, the need to identify anxiety is even greater. With better detection of anxiety, and greater understanding of how anxiety can impact cognitive functioning, more appropriate and tailored interventions can be implemented to support these vulnerable children.

## Data availability statement

The raw data supporting the conclusions of this article will be made available by the authors, without undue reservation.

## Ethics statement

The studies involving human participants were reviewed and approved by the La Trobe University Human Research Ethics Committee, Victorian Department of Education, and Catholic Education Melbourne. Written informed consent to participate in this study was provided by the participants' legal guardian/next of kin.

## Author contributions

HP: conceptualisation, formal analysis, investigation, methodology, project administration, and writing—original draft. CP: conceptualisation, resources, and writing—review and editing. SC: conceptualisation, methodology, resources, supervision, and writing—review and editing. All authors contributed to the article and approved the submitted version.

## Conflict of interest

The authors declare that the research was conducted in the absence of any commercial or financial relationships that could be construed as a potential conflict of interest.

## Publisher’s note

All claims expressed in this article are solely those of the authors and do not necessarily represent those of their affiliated organizations, or those of the publisher, the editors and the reviewers. Any product that may be evaluated in this article, or claim that may be made by its manufacturer, is not guaranteed or endorsed by the publisher.
