# Supplementary material for: The effect of anxiety on working memory and language abilities in elementary schoolchildren with and without Additional Health and Developmental Needs
Source: Front Psychol. 2022 Dec 15;13:1061212. doi: 10.3389/fpsyg.2022.1061212 (PMC9797981; doi:10.3389/fpsyg.2022.1061212)
Supplement: Supplementary file 1 [file Data_Sheet_1.pdf]

Supplemental Document

The Effect of Anxiety on Working Memory and Language Abilities in Elementary School Children With and Without Additional Health and Developmental Needs

Hayley E Pickering*, Carl Parsons, and Sheila G Crewther

*Corresponding Author

Hayley E Pickering

Department of Psychology, Counselling and Therapy,

La Trobe University, Melbourne VIC 3086, Australia

Email: h.pickering@latrobe.edu.au

Associated article can be found here:

https://www.frontiersin.org/articles/10.3389/fpsyg.2022.1061212/full

Table of Contents

Table S1: Diagnoses of AHDN Children 2

Alberta Language and Development Questionnaire Scoring 3

Table S3: Correlations 7

Table S4: Comparing AHDN and NT Groups, Excluded Language Disorders 8

References 9

Table S1

Diagnoses of Additional Health and Developmental Needs (AHDN) Children

**Diagnosis**	**N**	**% AHDN sample**
None	22	53.7
Autism Spectrum Disorder	6	14.6
Language Disorder	5	12.2
Attention-Deficit/Hyperactivity Disorder	4	9.8
Dyslexia/Specific Learning Disorder (reading)	1	2.4
Autism Spectrum Disorder + Language Disorder	1	2.4
Autism Spectrum Disorder + Language Disorder + Dyslexia	1	2.4
Undergoing Assessment	1	2.4

Note. $n = 41$.

Alberta Language and Development Questionnaire Scoring

The Alberta Language and Development Questionnaire (ALDeQ; Paradis et al., 2010) was designed to be a culture-free assessment of child language and development that is not specific to any one language. Whilst all children included in the current study spoke English as a first language, data included in the current study was part of a larger project that included children who may not speak English as their first language. Thus, questions are framed in relation to the child's first language. Although the ALDeQ is traditionally given as a structured interview, for the current study we adapted questions from the first two sections to be a parent-rated measure. The questions, possible answers, and associated scores are provided below. For each section, points are summed and divided by the number of questions, so total scores range between 0 (very poor development) and 1 (very good development).

Section A: Early Milestones

1. How old was your child when they first said a word (in any language)?
   i. Before 12 Months 6 points
   ii. 12-15 Months 6 points
   iii. 16-24 Months 4 points
   iv. After 24 Months 0 points
2. How old was your child when they first put words together in short sentences (in any language)?
   i. Before 24 Months (2 Years) 6 points
   ii. 25-30 Months (2-2½ Years) 4 points
   iii. After 30 Months (2½ Years) 0 points

Section B: Current Abilities in First Language

1. Compared to other children the same age as your child, how well does your child express themselves in their first language?
   i. Not well at all 0 points
   ii. A little less well 1 point

iii.	The same	2 points
iv.	Better	3 points

2. Compared to other children the same age as your child, how well does your child pronounce words in their first language?

i.	Not very clearly	0 points
ii.	Sometimes not clear	1 point
iii.	The same	2 points
iv.	Better	3 points

3. Can family or friends easily talk with your child in their first language?

i.	Very easily	3 points
ii.	Easy enough	2 points
iii.	Sometimes not easy	1 point
iv.	Always not easy	0 points

4. Compared to other children the same age as your child, is it hard for your child to produce correct sentences in their first language?

i.	Not hard	3 points
ii.	The same	2 points
iii.	A little hard	1 point
iv.	A lot hard	0 points

5. Are you satisfied with how your child speaks your language/their first language?

i.	Completely	3 points
ii.	Somewhat	2 points
iii.	A little	1 point
iv.	Not at all	0 points

Table S2

Partial correlations [95% CI] between all measures in AHDN and NT children

	RCPM	SCAS-P	Vis DS-F	Aud DS-F	Vis DS-B	Aud DS-B	PPVT	EVT	Elision	ALDeQ-E	ALDeQ-C
RCPM	-	.089<br>[-.237, .397]	.147<br>[-.181, .446]	.292<br>[-.031, .560]	.389*<br>[.079, .630]	.408**<br>[.102, .644]	.549***<br>[.278, .739]	.512***<br>[.230, .715]	.511***<br>[.229, .714]	.264<br>[-.061, .538]	.203<br>[-.125, .491]
SCAS-P	.013<br>[-.308, .331]	-	-.131<br>[-.433, .196]	.090<br>[-.237, .398]	-.175<br>[-.469, .153]	-.108<br>[-.413, .219]	.007<br>[-.313, .326]	-.162<br>[-.458, .166]	-1.07<br>[-.413, .220]	-.125<br>[-.428, .203]	-.207<br>[-.494, .121]
Vis DS-F	-.166<br>[-.461, .162]	.128<br>[-.200, .430]	-	.422**<br>[.118, .654]	.323*<br>[.004, .583]	.244<br>[-.082, .523]	.296<br>[-.026, .563]	.308<br>[-.013, .571]	.425**<br>[.122, .656]	.103<br>[-.224, .409]	.212<br>[-.116, .498]
Aud DS-F	-.017<br>[-.335, .304]	.191<br>[-.137, .481]	.469**<br>[.176, .686]	-	.383*<br>[.072, .626]	.032<br>[-.291, .348]	.343*<br>[.026, .597]	.264<br>[-.061, .538]	.340*<br>[.023, .595]	.164<br>[-.164, .460]	.171<br>[-.157, .465]
Vis DS-B	.268<br>[-.057, .541]	-.242<br>[-.521, .084]	.218<br>[-.109, .503]	.100<br>[-.227, .407]	-	.363*<br>[.049, .612]	.210<br>[-.118, .496]	.396*<br>[.087, .635]	.574***<br>[.312, .755]	.250<br>[-.076, .528]	.428**<br>[.126, .658]
Aud DS-B	.256<br>[-.069, .532]	-.203<br>[-.491, .125]	.340*<br>[.023, .595]	.176<br>[-.152, .469]	.631***<br>[.390, .791]	-	.233<br>[-.094, .514]	.395*<br>[.086, .635]	.310<br>[-.011, .573]	-.048<br>[-.362, .276]	.218<br>[-.109, .503]
PPVT	.157<br>[-.171, .454]	-.093<br>[-.401, .234]	.388*<br>[.078, .230]	.290<br>[-.033, .558]	.187<br>[-.141, .478]	.331*<br>[.013, .588]	-	.658***<br>[.428, .808]	.257<br>[-.068, .533]	.044<br>[-.280, .359]	.001<br>[-.319, .321]
EVT	-.078<br>[-.388, .248]	.015<br>[-.306, .333]	.122<br>[-.206, .425]	-.251<br>[-.528, .075]	.252<br>[-.074, .529]	.130<br>[-.120, .432]	.308<br>[-.013, .571]	-	.456**<br>[.160, .677]	.246<br>[-.080, .524]	.365*<br>[.051, .613]
Elision	.465<br>[.170, .683]	-.065<br>[-.377, .260]	.148<br>[-.180, .447]	.115<br>[-.213, .419]	.399*<br>[.091, .637]	.335<br>[.017, .591]	.304<br>[-.017, .568]	.280<br>[-.044, .550]	-	.222<br>[-.105, .506]	.391*<br>[.082, .632]
ALDeQ-E	-.057<br>[-.370, .268]	-.114<br>[-.418, .214]	.225<br>[-.102, .508]	.154<br>[-.174, .452]	.114<br>[-.214, .418]	.498**<br>[.212, .705]	.221<br>[-.117, .497]	-.009<br>[-.328, .312]	-.006<br>[-.325, .314]	-	.412**<br>[.106, .647]
ALDeQ-C	-.379*<br>[-.623, -.068]	-.194<br>[-.484, .134]	.179<br>[-.149, .472]	.108<br>[-.219, .413]	.119<br>[-.209, .423]	.303<br>[-.019, .568]	.388<br>[.078, .630]	.364*<br>[.050, .612]	-.034<br>[-.350, .289]	.272<br>[-.052, .545]	-

Note. Partial correlation controlling for age. Results for the AHDN group (n = 41, df = 38) below the diagonal, and results for the NT group (n = 41, df = 38) are above the diagonal. AHDN = Additional Health and Developmental Needs; NT = Neurotypical; RCPM = Raven's Coloured Progressive Matrices (Raven et al., 1998); SCAS-P = Spence Children's Anxiety Scale – Parent (Nauta et al., 2004); Vis = Visual; DS-F = Digit Span Forward; Aud = Auditory; DS-B = Digit Span Backward; PPVT = Peabody Picture Vocabulary Test (Dunn & Dunn, 2007); EVT = Expressive Vocabulary Test (Williams, 2007); ALDeQ = Alberta Language and Development Questionnaire (Paradis et al., 2010); E = Early; C = Current. Elision task Wagner et al., (2013).
$^{*}p$ = < .05, $^{**}p$ = < .01, $^{***}p$ = < .001.

Table S3

Comparing matched AHDN and NT groups, excluding children with a Language Disorder

	AHDN (n = 35)		NT (n = 35)		p	Effect Size, r
	M (SD)	Range	M (SD)	Range		
Age	6.975 (1.05)	5.05-9.05	6.95 (.99)	5.30-8.79	.972	.00
RCPM SS	108.71 (12.58)	89-139	109.00 (11.27)	92-133	.888	.02
Total SCAS-P	20.04 (14.49)	1-61	12.3 (7.72)	0-34	.021	.28
Vis DS-F	3.69 (1.05)	2-6	4.37 (1.06)	2-6	.009	.31
Aud DS-F	4.60 (.95)	2-7	4.91 (1.17)	2-8	.138	.18
Vis DS-B	2.54 (1.20)	0-5	3.29 (1.02)	0-5	.006	.33
Aud DS-B	2.46 (.98)	0-4	3.17 (.74)	2-5	.002	.37
PPVT SS	107.11 (12.02)	89-129	113.43 (10.46)	87-130	.029	.26
EVT SS	97.23 (10.22)	76-117	110.69 (10.72)	87-131	<.001	.55
Elision SS	8.11 (1.78)	4-12	10.69 (2.36)	7-16	<.001	.54
ALDeQ-Early	.88 (.17)	.33-1.00	.95 (.13)	.33-1.00	.038	.25
ALDeQ-Current	.65 (.26)	.07-1.00	.89 (.09)	.67-1.00	<.001	.54

Note. AHDN = Additional Health and Developmental Needs; NT = Neurotypical; RCPM = Raven's Coloured Progressive Matrices (Raven et al., 1998); SS = Standard Score (M = 100, SD = 15); SCAS-P = Spence Children's Anxiety Scale – Parent (Nauta et al., 2004); Vis = Visual; DS-F = Digit Span Forward; Aud = Auditory; DS-B = Digit Span Backward; PPVT = Peabody Picture Vocabulary Test (Dunn & Dunn, 2007); EVT = Expressive Vocabulary Test (Williams, 2007); ALDeQ = Alberta Language and Development Questionnaire (Paradis et al., 2010). Elision task from Wagner et al., (2013).

References

Dunn, L. M., & Dunn, D. M. (2007). *Peabody Picture Vocabulary Test* (4th ed.). Pearson.

Nauta, M. H., Scholing, A., Rapee, R. M., Abbott, M., Spence, S. H., & Waters, A. (2004). A parent-report measure of children's anxiety: Psychometric properties and comparison with child-report in a clinic and normal sample. *Behaviour Research and Therapy*, *42*(7), 813-839. https://doi.org/10.1016/s0005-7967(03)00200-6

Paradis, J., Emmerzael, K., & Duncan, T. S. (2010). Assessment of English language learners: Using parent report on first language development. *Journal of Communication Disorders*, *43*(6), 474-497. https://doi.org/10.1016/j.jcomdis.2010.01.002

Raven, J., Raven, J. C., & Court, J. H. (1998). *Manual for Raven's Progressive Matrices and Vocabulary Scales. Section 2: The coloured Progressive Matrices*. Oxford Psychologist Press.

Wagner, R. K., Torgesen, J. K., Rashotte, C. A., & Pearson, N. A. (2013). *Comprehensive Test of Phonological Processing* (Second ed.). Pro-ed.

Williams, K. T. (2007). *EVT-2: Expressive Vocabulary Test*. Pearson.
